# Posterior Reversible Encephalopathy Syndrome With Isolated Involving Infratentorial Structures

**DOI:** 10.3389/fneur.2018.00843

**Published:** 2018-10-09

**Authors:** Shuchun Ou, Lu Xia, Li Wang, Li Xia, Qin Zhou, Songqing Pan

**Affiliations:** Department of neurology, Renmin Hospital of Wuhan University, Wuhan, China

**Keywords:** brainstem, cerebellum, obstructive hydrocephalus, posterior reversible encephalopathy syndrome/hypertensive encephalopathy, spinal cord

## Abstract

Typical neuroimaging findings of posterior reversible encephalopathy syndrome include symmetrical white matter edema in subcortical white matter of bilateral occipital and parietal lobes, although variations do occur and more and more attention is being focused upon disease of infratentorial-isolated involved posterior reversible encephalopathy syndrome. In this article, we described 1 case of posterior reversible encephalopathy syndrome with isolated infratentorial brain involvement and reviewed the literature to identify an additional 36 cases in the PubMed database. We used various search terms, such as “brainstem/cerebella/spinal posterior reversible encephalopathy syndrome,” “brainstem/cerebella/spinal reversible posterior leukoencephalopathy syndrome,” “brainstem/cerebella/spinal hypertensive encephalopathy,” “infratentorial posterior reversible encephalopathy syndrome,” and “posterior reversible encephalopathy syndrome variant.” Then, we systematically analyzed the clinical and imaging characteristics of the 37 cases and found that posterior reversible encephalopathy syndrome with isolated involving infratentorial structures predominantly affect male patients compared with typical posterior reversible encephalopathy syndrome. The presence of extremely high blood pressure at onset is essential to the development of infratentorial-isolated involved posterior reversible encephalopathy syndrome. A relatively high rate of hydrocephalus and spinal cord involvement can be a distinctive feature of this kind of variant. Symptoms and outcomes are basically similar to typical posterior reversible encephalopathy syndrome.

## Highlights

1. This study described a rare variant of PRES that isolated involving infratentorial structures (IIPRES).
2. We found that IIPRES mostly occurs in males and the presence of extremely high blood pressure at onset is essential to the development of IIPRES. We also analyzed the features of spinal cord involvement and obstructive hydrocephalus in IIPRES.
3. Our purpose was to compare PRES contributing factors, imaging features and outcomes in infratentorial-predominant PRES vs. typical PRES with the goal of better understanding the natural history of IIPRES.

## Introduction

Posterior reversible encephalopathy syndrome (PRES) was first described as a reversible syndrome manifesting with acute headaches, altered mental status, seizures, and visual disturbances by Hinchey et al. (1). Magnetic resonance imaging (MRI) usually implies vasogenic edema predominantly locating in subcortical white matter of bilateral occipital and parietal lobes, the exact pathogenesis of which has yet to be explained (1–5). Severe hypertension, renal dysfunction, eclampsia/pre-eclampsia, and the use of immunosuppressive drugs are thought to be main etiologies (1–3, 5). With aggressive antihypertensive treatment, most patients can achieve complete resolution both in the clinic and radiology.

Vasogenic edema in the typical parietal or occipital regions occur in more than 90% of PRES patients (3, 6). In recent years, new terms, such as central-variant PRES and brainstem variant PRES have appeared to widen the spectrum of atypical PRES (3, 6–9). A previous study reported that an atypical pattern of involvement of the brainstem, basal ganglia, and periventricular white matter with sparing of the typical frontal, parietal, and occipital cortexes occur in approximately 4% of PRES patients (9). However, there are no published reports which systematically analyze isolated involvement infratentorial brain to our knowledge. Here we describe a variant of PRES exclusively involving infratentorial areas (IIPRES), with the supratentorial structures completely spared, and investigate the different clinical and radiological characteristics of this condition compared to typical PRES.

## Patients and methods

### Patients

We describe a patient with isolated infratentorial brain involvement seen at the Neurology Department of RenMin Hospital of Wuhan University, and identified an additional 36 cases (18 articles) (7, 10–26) in the PubMed database from 2000 to date using various search terms related to “infratentorial-predominant PRES,” “PRES variant,” “hypertensive encephalopathy” “brainstem hypertensive encephalopathy,” and “PRES isolated involvement of brainstem/cerebella.”

#### Case report

**Figure 1:**
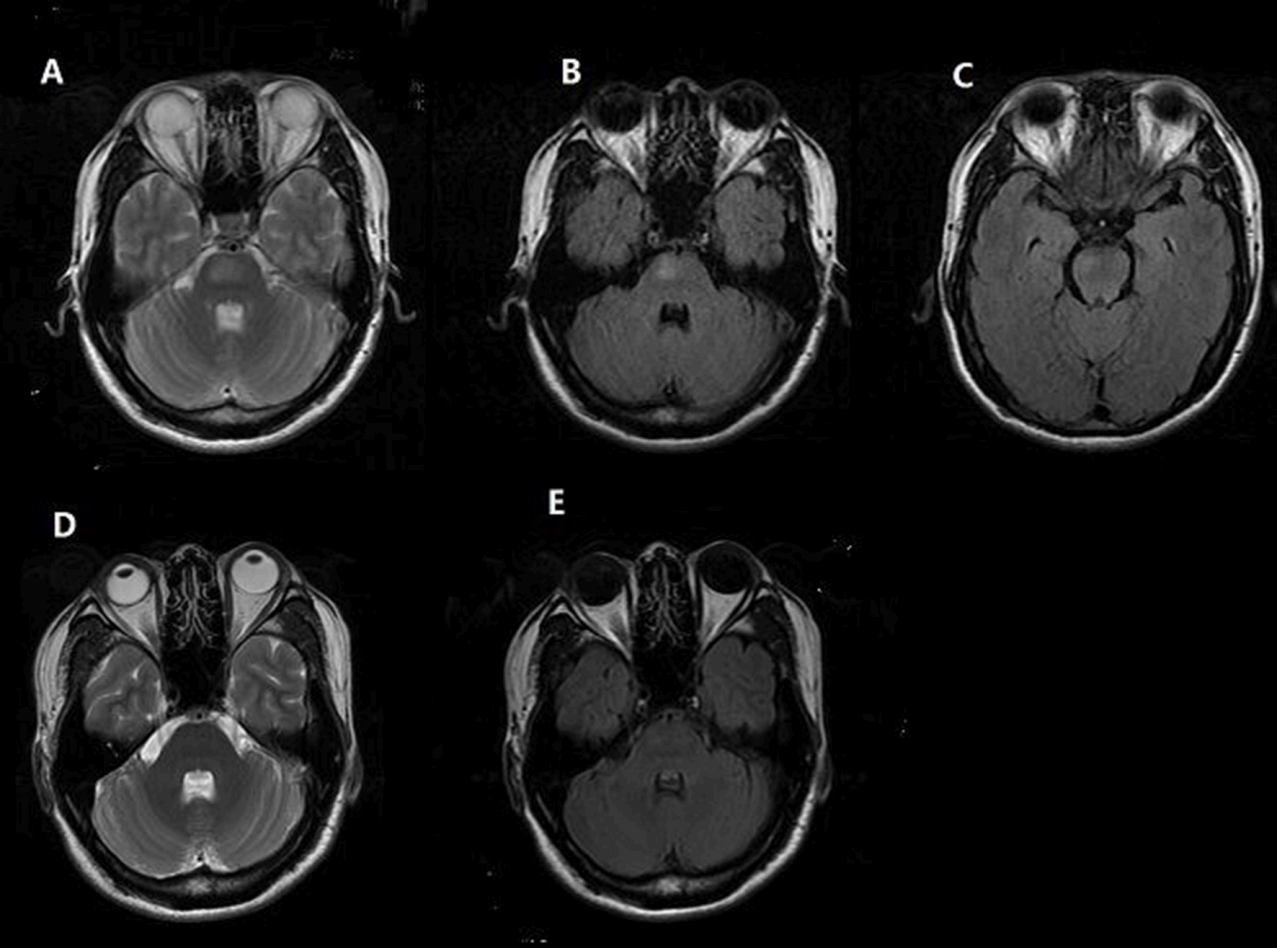
Brain MRI finding. Patient 1, **(A,B)** Axial T2-weighted and fluid attenuated inversion recovery (FLAIR) MR image reveals hyperintense lesion in pons. **(C)** No abnormal signal in the parietal and occipital lobes was found. **(D,E)** Follow-up MRI at 1 month shows complete resolution of the hyperintensity in the brainstem.

A 41-year-old man presented with a headache for 1 day that predominantly affected the prefrontal and occipital regions. Persistent headache brought him into the hospital. He had no medical history of headache and hypertension and there was no history of head or neck trauma. Blood pressure was 200/140 mmHg on admission. He had no alterations in consciousness or visual symptoms. There were no hyperreflexia, ataxia, or other abnormal neurological examination results. Head computer tomography (CT) revealed no significant abnormities. Laboratory examinations showed a urine protein level of 2+ and 24-h urine protein was 1.06 g. Urine potassium and sodium were 39.25 and 315 mmol/24 h, respectively, demonstrating the impairment of renal function. Serum potassium was 3.23 mmol/L and serum sodium level was normal. The signal in the pons was increased in T2-weighted and fluid attenuated inversion recovery (FLAIR) image but was normal-intensity in T1-weighted images (Figures 1A,B). There were no abnormal signals in the parietal and occipital lobes (Figure 1C). Unfortunately, the patient did not receive diffusion-weighted imaging (DWI) on admission.

In consideration of his mild clinical manifestations (the severity of symptoms did not match the extent of his lesion) and normal neurological examinations, we excluded the diagnosis of brainstem infarction; also, the normal concentration of serum sodium may help to rule out the diagnosis of pontine myelinolysis. Then, we came to the diagnosis of PRES and initiated aggressive antihypertensive treatment with Irbesartan, Hydrochlorothiazide, Nifedipine, and Spironolactone. His symptoms completely resolved on the third day. A month later, repeated MRI showed complete resolution of the abnormalities in the brainstem (Figures 1D,E). Thus, the rare “reversible” characteristic of lesions following antihypertensive treatment confirmed the diagnosis of PRES.

This study was carried out in accordance with the approval of the Ethics Review Committee of Wuhan University Renmin Hospital. The subject gave written informed consent for the publication of clinical details in accordance with the Declaration of Helsinki.

### Methods

We include articles published in English describing patients with sufficient clinical and imaging detail. All cases referred to were without typical parietooccipital abnormalities. We regard clinical and radiological features as being present if they were described and absent if not mentioned.

## Results

We found that 18 reports of 36 patients met our inclusion criteria. With the addition of our patient, a total of 37 cases were included in our descriptive analysis. The main results were presented in Table 1.

**Table 1:** Demographics, clinical and radiological characteristics of 37 patients.

**Case**	**Gender**	**Age**	**HTN**	**Renal**	**BP(MAP)**	**Headache**	**Mental**	**Visual**	**Seizure**	**Location**	**Hydrocephalus**	**Treatment**
1	M	41	–	+	200/140(160)	+	–	–	–	Brainstem	–	Antihypertension
2^10^	M	62	+	–	180/100(127)	+	+	–	+	Brainstem	–	Antihypertension
3^11^	M	50	+	–	–	+	–	+	–	Brainstem	–	Antihypertension
4^12^	M	49	–	+	202/138(159)	–	+	–	–	Brainstem	–	Antihypertension
5^13^	M	60	–	–	220/150(173)	–	+	–	–	Brainstem	–	Antihypertension
6^14^	F	39	+	+	190/110(137)	+	–	+	–	Brainstem	–	Antihypertension
7^15^	F	67	–	+	204/106(138)	–	+	–	–	Brainstem	–	Antihypertension
8^16^	M	37	–	–	210/140(163)	–	–	–	–	Brainstem	–	Antihypertension
9^16^	M	51	+	–	150/110(123)	–	–	–	–	Brainstem	–	Antihypertension
10^16^	M	51	–	+	250/130(170)	–	+	–	–	Brainstem	–	Antihypertension
11^16^	M	52	+	–	230/140(170)	–	–	+	–	Brainstem	–	Antihypertension
12^16^	M	76	–	–	210/180(190)	–	+	–	–	Brainstem brainstem	–	Antihypertension
13^17^	M	32	+	+	220/140(167)	+	+	–	+	Brainstem	–	Antihypertension
14^18^	F	84	+	–	238/128(165)	–	+	+	–	Cerebellum	–	Antihypertension
15^7^	M	33	+	+	230/120(156)	+	–	–	–	Cerebellum	–	Antihypertension
16^7^	M	21	–	–	210/140(163)	+	–	–	–	Cerebellum	–	Antihypertension
17^7^	M	7	–	–	150/110(123)	+	+	–	+	Cerebellum	–	Antiepileptic
18^7^	F	7	–	+	270/220(237)	+	+	–	+	Cerebellum	–	Antihypertension
19^7^	F	37	+	+	210/150(170)	+	–	+	–	Cerebellum	–	Antihypertension
20^7^	M	11	–	+	206/148(167)	+	–	+	–		+	Antihypertension;EVD
										Cerebellum		Antihypertension;
21^7^	F	49	–	–	280/160(200)	–	+	–	–	Cerebellum	+	Antihypertension;EVD
22^7^	M	52	+	+	240/160(187)	+	+	–	–	Cerebellum	+	Antihypertension;EVD
23^7^	M	26	+	+	175/110(132)	–	+	+	–	Cerebellum	+	Antihypertension;EVD
24^19^	M	52	–	+	242/145(177)	+	–	–	–	Cerebellum	+	Antihypertension
25^20^	M	13	–	+	260/150(187)	–	+	–	–	Cerebellum	+	Antihypertension;EVD
26^21^	M	32	+	–	250/140(177)	+	–	–	–	Brainstem;	+	Antihypertension
27^22^	M	48	+	–	248/147(181)	–	+	–	–	Cerebellum	+	Antihypertension;EVD
										Brainstem; cerebellum		
28^22^	M	49	+	+	245/148(180)	–	+	–	–	Brainstem; cerebellum	+	Antihypertension;EVD
										Brainstem; cerebellum		
29^22^	M	58	+	–	230/122(158)	+	–	–	–	Brainstem; cerebellum	+	Antihypertension
										Brainstem; cervical cord		
30^23^	F	10	–	+	141/105(117)	+	+	–	–	Brainstem; cervical cord	–	Antihypertension
										Brainstem; cervical cord		
31^21^	M	42	–	+	190/110(137)	+	–	–	–	Brainstem; cervical cord	–	Antihypertension;
										Brainstem; cervical cord		Haemodialysis
32^10^	M	59	+	–	210/108(142)	–	+	–	+	Brainstem; cerebellum; cervical cord	–	Antihypertension
33^24^	F	9	–	+	225/110(148)	+	–	+	–	brainstem; cervical cord	–	Antihypertension
34^25^	M	25	+	+	225/159(181)	+	–	+	–	Brainstem; cervical cord	–	Antihypertension
35^25^	F	44	–	–	240/140(173)	+	+	+	–	Brainstem; cervical cord	–	Antihypertension
36^26^	F	26	–	–	210/105(140)	+	–	–	+	Brainstem; cervical cord	–	Antihypertension
37^25^	F	14	–	+	85/145(158)	+	+	+	–	Brainstem; cerebellum; cervical cord	–	Antihypertension

*HTN, hypertension; BP, blood pressure; MAP, mean arterial pressure; EVD, external ventricular drainage. +, present, –, absent. Patient 1 is reported in this article*.

### Clinical characteristics

Twenty six patients were male (70.3%) and 11 were female (29.7%), with an average age of 39.9 ± 19.8 years (range, 7–84 years) and 7 were children. All cases but one whose blood pressure didn't record had severe, acute hypertension. Mean blood pressure was 216/135 mmHg (MAP 162 ± 24.7 mmHg). Seventeen out of 37 patients had a clear medical history of hypertension (45.9%), 19 had renal impairment (51.4%), and 2 (5.4%, 7 years and 10 years, respectively) were receiving chemotherapy for leucocythemia. The most common symptom was headache (22/37, 59.5%), followed by altered metal status (20/37, 54.1%), vomiting (14/37, 37.8%), visual disturbance (11/37, 29.7%), and seizure (6/37, 16.2%) in a descending order. Other rare symptoms included difficulty in walking (5/37, 13.5%), ataxia (4/37, 10.8%), speech disorder (3/37, 8.1%), vertigo (3/37, 8.1%), and urinary urgency (1/37, 2.7%).

### Imaging findings

All patients had hyperintensity signals on T2-weighted (T2W) and fluid-attenuated inversion recovery (FLAIR) images in the infratentorial brain. Isolated involvement of brainstem and cerebellum were 14 (14/37, 37.8%) and 12 (12/37, 32.4%), respectively. Ten (10/37, 27.0%) of the patients raised a complication of obstructive hydrocephalus and 7 of them received external ventricular drainage (EVD). Six patients (6/37, 16.2%) involved the spinal cord and the longest lesion was observed from the lower pons to the Th8 level.

### Treatment and outcome

The clinical manifestations, presented acute or subacute, achieved a favorable resolution among majority patients with progressive antihypertensive treatment and EVD, as well as the imaging findings. In addition to 6 patients (6/37, 16.2%) with persisted imaging abnormalities, follow-up MRI showed significant improvement as early as 5 days and as late as 11 months after the initial scan.

## Discussion

We identified several differences of clinical features between our case and typical PRES. Our series is male-dominant (male 70.3 vs. 13%) compared to typical PRES reported in initial literature by Hinchey et al. (1). All recorded cases (except one which did not have a record) had acute and extremely high hypertension with a mean up to 216/135 mmHg (MAP 162 ± 24.7 mmHg), a much higher level compared to the mean of 165/97 mmHg in the article published by Hinchey. The proportion of normotension/hypotension in patients with typical PRES might imply the possibility of acute and severe hypertension is an essential condition for IIPRES (2, 7). Similar to typical PRES, a medical history of hypertension and renal impairment takes up a large proportion as etiology (45.9 and 51.3%, respectively). Except for the extremely low proportion of immunosuppressive therapy (5.4 vs. 53%) in our series, we did not encounter any patients in pregnant or pre-eclamptic/eclamptic conditions.

**Figure 2:**
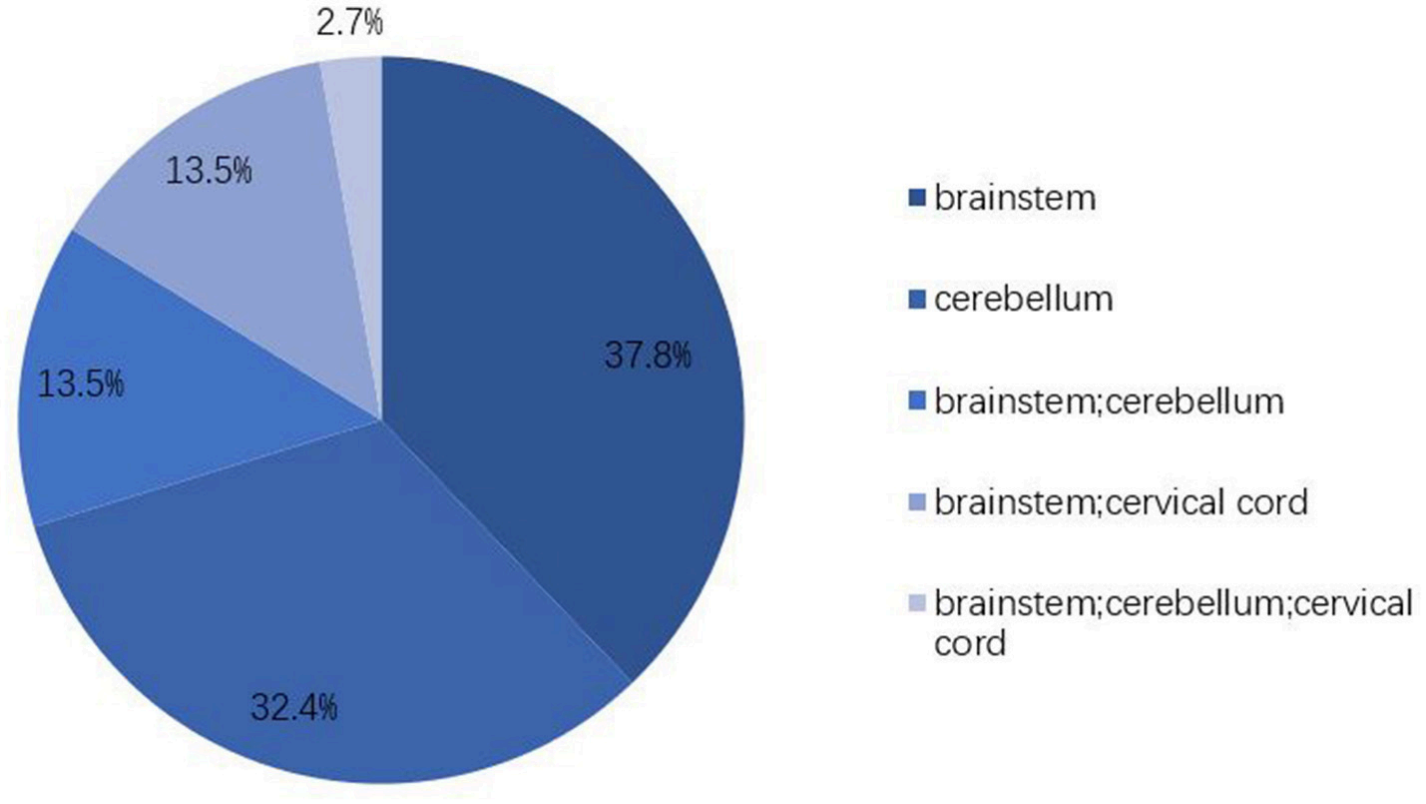
Lesion distributions. Vasogenic edema in IIPRES affects predominantly the brainstem (67.5%) and cerebellum (48.6%). Cervical cord involvement takes up 16.2%. Extensive involvement of posterior cranial fossa is very rare (2.7%).

PRES with atypical radiology involving the frontal and temporal lobes, basal ganglia, brainstem, cerebellum, and other cerebral areas has been reported in recent years (3, 4, 6, 7, 27). These atypical distributions have a higher rate than we thought (21). It is reported that the incidence of brainstem involvement in PRES was in the range of 13–18.4% and cerebellum in ~30%, almost always accompanied by abnormalities in typical regions (3, 4, 6). However, there is no systematic description of PRES that isolated involving infratentorial brain. Figure 2 clearly shows that the brainstem (37.8%) and cerebellum (32.4%) are easily affected in a solitary manner and that extensive involvement of posterior cranial fossa (2.7%) is very rare. There is no association between lesion location and clinical manifestation. Headache and altered mental status were evident in most patients in our series, as well as in typical PRES, but the incidence of visual disturbance (29.7 vs. 66.7%) and seizure (16.2 vs. 73.3%) in IIPRES was significantly lower than typical PRES (1, 3, 5). Researchers have tried to seek a relationship between the occipital involvement of PRES and the presentation of visual disturbance but have failed to do so (28). Other focal presentations, such as weakness of limbs, ataxia, or aphasia, presented in 5–15% of documented patients were also been reported in our series (2).

Another hallmark in our series is of spinal cord involvement (16.2%). One article systematically described a variant of PRES with spinal cord involvement (PRES-SCI) (25). The clinical characteristics were found to resemble to our current findings, and all PRES-SCI are complicated by brainstem lesions; thus, some physicians feel that this represents part of the brainstem variant of PRES (20, 25, 29). The shared vertebrobasilar blood-supply system might explain the susceptibility of edema in the spinal cord while a relatively dense sympathetic innervations of spinal cord probably responsible for the low incidence of PRES-SCI (25, 29). This type of protective mechanism may indicate a more serious condition, but in fact, there is no corresponding symptom of spinal lesion and the outcome is basically similar to typical PRES.

In addition, there were 10 patients (27.0%) accompanied by obstructive hydrocephalus in our series which is not usually seen in typical PRES. Hydrocephalus occurred in 30–54.4% patients of PRES mainly involving the brainstem and cerebellum (7, 8). This may be relevant to the distinctive anatomical characteristics of the infratentorial structures of brain. A retrospective study pointed out that only half of the patients with hydrocephalus require EVD placement; the majority had a full resolution with adequate management of blood pressure (22).

The exact mechanism of PRES still remains controversial and two leading theories attempt to explain the pathophysiology of this condition. One hypothesis is that blood flow in the brain is maintained by the cerebrovascular autoregulation and an increase in blood pressure exceeding the cerebrovascular autoregulatory limits leads to vasodilation following a disruption of the blood-brain barrier and extravasation of plasma and macromolecules, presenting vasogenic rather than cytotoxic edema in MRI imaging. One research study demonstrated that mean blood pressure plays a vital role in the blood-brain barrier disruption in the evolution of a possible model of PRES (30). However, the endothelial theory rests on the assumption that the initial factor is endothelial damage secondary to systemic toxicity in immunosuppressive therapy, eclampsia, and sepsis. These pathological conditions damage by direct cytotoxicity of immunosuppressants such as calcineurin inhibitors which are known to cause endothelial cell injury, or indirectly by the induction of excessive cytokines release which can activate endothelial cells to secrete vasoactive factors, increase vascular permeability, and lead to interstitial brain edema (15).

Several articles pointed out that the lower density of sympathetic innervations in the vertebrobasilar system, an essential factor for the control of cerebrovascular autoregulation (presumably protecting the brain from marked increases in intravascular pressure, such as with severe hypertension), might be attributed to the high incidence of reversible vasogenic edema in posterior zone (1, 7, 9, 27). There is no clear explanation for isolated involvement of infretentorial territories thus far. Some studies have demonstrated that mild elevation of hypertension predominantly involved the supratentorial areas with little or no involvement of the infratentorial areas while severe elevation of hypertension are likely to cause vasogenic edema in the brain stem, basal ganglia and cerebellum (5, 31). This is consistent with our finding of extraordinarily high blood pressure correlated with the development of IIPRES. Some others have speculated that this variant may have a potential relationship with individual variation (28). There are also reports in the literature proposing that great fluctuations of blood pressure rather than absolute increase as well as the rising proportion and rising speed probably play a key role in the development of edema (2).

Precisely because of the characteristic of vasogenic edema, PRES is commonly thought to be a benign process with good reversibility both in clinic and imaging presentation. However, with prompt and appropriate treatment, not all patients achieved complete resolution and permanent damage existed either on clinical presentation or imaging, or both, in IIPRES. Persistent lesions on imaging were found in 6 patients with the longest follow-up exceeding 1 year. A multicenter study showed that the brainstem lesions showed less reversibility compared to typical cortical and subcortical PRES lesions (32). The poor correlation between clinical outcome and the presence of hemorrhage and diffusion restriction has been found too (23). A longer follow-up may be necessary to identify the long-term outcome of IIPRES.

The differential diagnosis includes central pontine myelinolysis, brainstem infarction, brainstem glioma, multiple sclerosis. The main identifications of IIPRES are vasogenic edema presented in imaging and the reversibility of clinic and imaging with aggressive antihypertensive treatment.

Our study has several limitations given its retrospective nature. First, we collected literature from 2000 until present and may therefore have missed earlier publications. Furthermore, we only worked with publications written in English. This may have led to an underestimation of the incidence of PRES variants. Second, it was difficult for us to perform a comprehensive collection of information such as the results of laboratory examinations, CSF and fundus examination. Finally, we did not carry out statistical analysis due to the relatively small number of patients. To further address these issues, a pooled analysis of multicenter studies is necessary and more efforts should be taken to investigate the distinctive characteristics of IIPRES.

## Conclusions

This article comprehensively introduces and analyses the characteristics of IIPRES. Our aim is to widen the spectrum of PRES and raise the awareness of IIPRES, providing clinicians with diverse clues with which to diagnose lesions located in infratentorial brain. Clinicians should heighten the awareness of IIPRES and take appropriate management timely in order to obtain a good prognosis.

## Author contributions

SO study concept and design. LuX study design. LW data analysis. LiX and QZ: picture editing. SP study supervision. All authors read and approved the final manuscript.

### Conflict of interest statement

The authors declare that the research was conducted in the absence of any commercial or financial relationships that could be construed as a potential conflict of interest.

## References

[1] Hinchey J Chaves C Appignani B Breen J Pao L Wang A. A reversible posterior leukoencephalopathy syndrome. N Engl J Med. (1996) 334:494–500. 10.1056/NEJM199602223340803 8559202

[2] Fugate JE Rabinstein AA. Posterior reversible encephalopathy syndrome: clinical and radiological manifestations, pathophysiology, and outstanding questions. Lancet Neurol. (2015) 14:914–25. 10.1016/S1474-4422(15)00111-8 26184985

[3] Bartynski WS Boardman JF. Distinct imaging patterns and lesion distribution in posterior reversible encephalopathy syndrome. AJNR Am J Neuroradiol. (2007) 28:1320–7. 10.3174/ajnr.A0549 17698535 PMC7977645

[4] Fugate JE Claassen DO Cloft HJ Kallmes DF Kozak OS Rabinstein AA. Posterior reversible encephalopathy syndrome: associated clinical and radiologic findings. Mayo Clin Proc. (2010) 85:427–32. 10.4065/mcp.2009.0590 20435835 PMC2861971

[5] Fischer M Schmutzhard E. Posterior reversible encephalopathy syndrome. J Neurol. (2017) 264:1608–16. 10.1007/s00415-016-8377-8 28054130 PMC5533845

[6] McKinney AM Short J Truwit CL McKinney ZJ Kozak OS SantaCruz KS. Posterior reversible encephalopathy syndrome: incidence of atypical regions of involvement and imaging findings. AJR Am J Roentgenol. (2007) 189:904–12. 10.2214/AJR.07.2024 17885064

[7] Li D Lian L Zhu S. Isolated cerebellar involvement in posterior reversible encephalopathy syndrome. J Neurol Sci. (2015) 357:101–5. 10.1016/j.jns.2015.07.004 26163418

[8] Cruz-Flores S de Assis Aquino Gondim F Leira EC. Brainstem involvement in hypertensive encephalopathy: clinical and radiological findings. Neurology (2004) 62:1417–9. 10.1212/01.WNL.0000120668.73677.5F 15111687

[9] McKinney AM Jagadeesan BD Truwit CL. Central-variant posterior reversible encephalopathy syndrome: brainstem or basal ganglia involvement lacking cortical or subcortical cerebral edema. AJR Am J Roentgenol. (2013) 201:631–8. 10.2214/AJR.12.9677 23971457

[10] Chen TY Lee HJ Wu TC Tsui YK. MR imaging findings of medulla oblongata involvement in posterior reversible encephalopathy syndrome secondary to hypertension. AJNR Am J Neuroradiol. (2009) 30:755–7. 10.3174/ajnr.A1337 18854436 PMC7051761

[11] Karakis I Macdonald JA Stefanidou M Kase CS. Clinical and radiological features of brainstem variant of hypertensive encephalopathy. J Vasc Interv Neurol. (2009) 2:172–6. 22518250 PMC3317337

[12] Ho TH Tsai CL Hsu YD Lee JT Yang FC Hsu CC. Posterior reversible encephalopathy syndrome mimicking brainstem infarction: a dilemma. Acta Neurol Taiwan (2016) 25:56–59. 27854093

[13] Gamanagatti S Subramanian S. Hypertensive encephalopathy: isolated pons involvement mimicking central pontine myelinolysis. Korean J Radiol. (2006) 7:218–9. 10.3348/kjr.2006.7.3.218 16969054 PMC2667606

[14] Yerdelen D Giray S Tan M Yildirim T. Hypertensive encephalopathy with atypical MRI leukoencephalopathy affecting brain stem and cerebellum. Acta Neurol Belg. (2009) 109:142–5. 19681447

[15] Ono Y Manabe Y Hamakawa Y Murakami T Omori N Hayashi Y. Localized lesions on MRI in a case of hypertensive brainstem encephalopathy. Intern Med. (2005) 44:1002–5. 10.2169/internalmedicine.44.1002 16258222

[16] Moon SN Jeon SJ Choi SS Song CJ Chung GH Yu IK. Can clinical and MRI findings predict the prognosis of variant and classical type of posterior reversible encephalopathy syndrome (PRES)? Acta Radiol. (2013) 54:1182–90. 10.1177/0284185113491252 23858507

[17] Osman Y Imam YZ Salem K Al-Hail H Uthman B Deleu D. Isolated brainstem involvement in a patient with hypertensive encephalopathy. Case Rep Neurol Med. (2013) 2013:540947. 10.1155/2013/540947 23533856 PMC3600275

[18] Ho TH Kao HW Chen SJ Lee JT Hsu YD. Hypertensive brainstem encephalopathy mimicking central pontine myelinolysis: a potential pitfall. Am J Emerg Med. (2016) 34:1918 e5–7. 10.1016/j.ajem.2016.02.040 26935223

[19] O'Riordan S McGuigan C Stevens J Chapman N Ball J. Reversible hypertensive cerebellar encephalopathy and hydrocephalus. J Neurol Neurosurg Psychiatry (2007) 78:1008–9. 10.1136/jnnp.2006.107672 17332051 PMC2117878

[20] Ettinger N Pearson M Lamb FS Wellons JC III. Pediatric posterior reversible encephalopathy syndrome presenting with isolated cerebellar edema and obstructive hydrocephalus. J Neurosurg Pediatr. (2014) 14:344–7. 10.3171/2014.6.peds13553 25062302 PMC4332559

[21] Lee JJ Cho S Park JM Park KI. A case of hypertensive brainstem encephalopathy presenting with severe headache and unilateral hearing loss. J Neurol Sci. (2015) 355:211–2. 10.1016/j.jns.2015.05.040 26055310

[22] Kumar A Keyrouz SG Willie JT Dhar R. Reversible obstructive hydrocephalus from hypertensive encephalopathy. Neurocrit Care (2012) 16:433–9. 10.1007/s12028-011-9663-z 22234407

[23] Shimizu Y Tha KK Iguchi A Cho Y Yoshida A Fujima N. Isolated posterior fossa involvement in posterior reversible encephalopathy syndrome. Neuroradiol J. (2013) 26:514–9. 10.1177/197140091302600504 24199811 PMC4202824

[24] Yis U Karaoglu P Kurul SH Soylu A Cakmakci H Kavukcu S. Posterior reversible leukoencephalopathy syndrome with spinal cord involvement in a 9-year-old girl. Brain Dev. (2016) 38:154–7. 10.1016/j.braindev.2015.07.001 26220877

[25] de Havenon A Joos Z Longenecker L Shah L Ansari S Digre K. Posterior reversible encephalopathy syndrome with spinal cord involvement. Neurology (2014) 83:2002–6. 10.1212/WNL.0000000000001026 25355822

[26] Hou X Xu J Chen Z Li G Jiang H. Posterior reversible encephalopathy syndrome with involvement of the cervical cord and medulla: a case report. J Clin Diagn Res. (2015) 9:CD01–2. 10.7860/JCDR/2015/10756.5376 25737981 PMC4347072

[27] Fitzgerald RT Samant RS Kumar M Van Hemert R Angtuaco EJ. Features of infratentorial-predominant posterior reversible encephalopathy syndrome. Acta Neurol Belg. (2015) 115:629–34. 10.1007/s13760-015-0431-2 25605260 PMC4510038

[28] Gupta V Bhatia V Khandelwal N Singh P Singhi P. Imaging findings in pediatric posterior reversible encephalopathy syndrome (PRES): 5 years of experience from a tertiary care center in India. J Child Neurol. (2016) 31:1166–73. 10.1177/0883073816643409 27071468

[29] Wu TY Wei DY Jordan A Kenedi C Smith AD Kilfoyle DH. Reversible hypertensive encephalomyelopathy - the spinal variant of the posterior reversible encephalopathy syndrome. N Z Med J. (2015) 128:65–8. 26101119

[30] Marrone LCP Martins WA Borges MT Rossi BC Brunelli JPF Vedana VM. Posterior reversible encephalopathy syndrome: clinical differences in patients with exclusive involvement of posterior circulation compared to anterior or global involvement. J Stroke Cerebrovasc Dis. (2016) 25:1776–80. 10.1016/j.jstrokecerebrovasdis.2016.03.042 27103268

[31] Li R Mitchell P Dowling R Yan B. Is hypertension predictive of clinical recurrence in posterior reversible encephalopathy syndrome? J Clin Neurosci. (2013) 20:248–52. 10.1016/j.jocn.2012.02.023 23219827

[32] Pande AR Ando K Ishikura R Nagami Y Takada Y Wada A. Clinicoradiological factors influencing the reversibility of posterior reversible encephalopathy syndrome: a multicenter study. Radiat Med. (2006) 24:659–68. 10.1007/s11604-006-0086-2 17186320

